# Deciphering Underlying Drivers of Disease Suppressiveness Against Pathogenic *Fusarium oxysporum*

**DOI:** 10.3389/fmicb.2019.02535

**Published:** 2019-11-12

**Authors:** Yannan Ou, C. Ryan Penton, Stefan Geisen, Zongzhuan Shen, Yifei Sun, Nana Lv, Beibei Wang, Yunze Ruan, Wu Xiong, Rong Li, Qirong Shen

**Affiliations:** ^1^Jiangsu Provincial Key Lab for Solid Organic Waste Utilization, National Engineering Research Center for Organic-based Fertilizers, Jiangsu Collaborative Innovation Center for Solid Organic Waste Resource Utilization, Nanjing Agricultural University, Nanjing, China; ^2^Center for Fundamental and Applied Microbiomics, Biodesign Institute, College of Integrative Sciences and Arts, Arizona State University, Mesa, AZ, United States; ^3^Department of Terrestrial Ecology, Netherlands Institute of Ecology (NIOO-KNAW), Wageningen, Netherlands; ^4^Hainan Key Laboratory for Sustainable Utilization of Tropical Bioresources, College of Tropical Crops, Hainan University, Haikou, China; ^5^Ecology and Biodiversity Group, Department of Biology, Institute of Environmental Biology, Utrecht University, Utrecht, Netherlands

**Keywords:** *Fusarium oxysporum*, disease-conducive soil, disease-suppressive soil, microbiome, invasion resistance

## Abstract

Soil-borne diseases, especially those caused by fungal pathogens, lead to profound annual yield losses. One key example for such a disease is Fusarium wilt disease in banana. In some soils, plants do not show disease symptoms, even if the disease-causing pathogens are present. However, the underlying agents that make soils suppressive against Fusarium wilt remain elusive. In this study, we aimed to determine the underlying microbial agents governing soil disease-suppressiveness. We traced the shift of microbiomes during the invasion of disease-causing *Fusarium oxysporum* f. sp. *cubense* in disease-suppressive and disease-conducive soils. We found distinct microbiome structures in the suppressive and conducive soils after pathogen invasion. The alpha diversity indices increased (or did not significantly change) and decreased, respectively, in the suppressive and conducive soils, indicating that the shift pattern of the microbiome with pathogen invasion was notably different between the suppressive and conductive soils. Microbiome networks were more complex with higher numbers of links and revealed more negative links, especially between bacterial taxa and the disease-causing *Fusarium*, in suppressive soils than in conducive soils. We identified the bacterial genera *Chryseolinea*, *Terrimonas*, and *Ohtaekwangia* as key groups that likely confer suppressiveness against disease-causing *Fusarium*. Overall, our study provides the first insights into agents potentially underlying the disease suppressiveness of soils against Fusarium wilt pathogen invasion. The results of this study may help to guide efforts for targeted cultivation and application of these potential biocontrol agents, which might lead to the development of effective biocontrol agents against Fusarium wilt disease.

## Introduction

Soils provide essential ecosystem services by, for instance, supporting plant growth. Plants are at the base of human nutrition. However, plant performance depends not only on nutrients and other abiotic factors but also on intimate interactions with (soil) biota (Oerke, 2006). Among the most prominent agents affecting plant performance are microbial pathogens (Oerke and Dehne, 2004; Oerke, 2006). However, pathogens are not able to inhibit plant performance in disease-suppressive soils. These soils naturally suppress crop disease even in the presence of the pathogen, in the presence of the host plant, and under favorable environmental conditions (Baker and Cook, 1974; Roget, 1995; Mazzola, 2004; Weller et al., 2007). Disease suppressive soils have been observed for various diseases (Kyselková et al., 2009; Mendes et al., 2011; Rosenzweig et al., 2012; Penton et al., 2014; Cha et al., 2016; Xiong et al., 2017). A thorough understanding of natural disease suppression, thought to be characteristic of many undisturbed soils, and may lead to sustainable alternative pathogen control strategies (Weller et al., 2002; Kinkel et al., 2011). Microorganisms are the main drivers of soil suppressiveness (Garbeva et al., 2004), and previous studies have uncovered microorganisms that are involved in disease suppression (Mendes et al., 2011; Cha et al., 2016). However, the identity of specific microbes and the underlying mechanisms that respond to the introduction and establishment of many pathogens have not been determined. Hence, further insight into the interactions between the pathogen and suppressive soil microbes, or the whole community, will provide further insights into the mechanisms underlying soil suppressiveness.

Among soil-borne pathogens, fungi arguably cause the majority of crop losses and concomitant yield reductions (Fisher et al., 2012). This property is especially true for pathogenic fungi within the *Fusarium oxysporum* complex, comprised of over 100 identified *formae speciales* that together affect hundreds of plant species and can persist in soil for years (Gerlach and Nirenberg, 1982). In this study, we used Fusarium wilt of banana as a model system. Banana Fusarium wilt is caused by the fungal pathogen *F. oxysporum* f. sp. *cubense* (Foc), which poses a serious threat to the banana industry and has resulted in profound economic damage in areas producing the most economically prominent and widespread banana variety, Cavendish (Ploetz, 2015). To date, this disease cannot be controlled with classical approaches, including pesticides (Ploetz, 2015). Irrigation leads to pathogen invasion (Dita et al., 2018), and the common banana monocropping systems are prone to pathogen accumulation (Shen et al., 2018). However, in some cases, soil disease suppressiveness can also be induced by continuous cultivation of a susceptible host plant, which is attributed to the soil immune responses by enrichment and activity-specific pathogen-suppressive microbes (Kinkel et al., 2011). Indeed, disease suppressive and conducive soils harbor unique microbial communities (Shen et al., 2015; Deltour et al., 2017; Bowen et al., 2019). Thus, the efficient control of Fusarium wilt disease might be hidden in disease suppressive soil microbiomes, and it is therefore essential to understand the soil microbial taxa and entire microbiome compositions that might induce suppressiveness. This knowledge will provide a foundation for potential manipulation of the soil microbial community to sustainably suppress the disease.

Based on recently identified suppressive and conducive soils to Fusarium wilt disease, we used Illumina metabarcoding to trace changes in microbiome composition upon pathogen invasion over time. We aimed to identify potential microbial agents that induce soil suppressiveness against Fusarium wilt disease by investigating expected changes in the microbiome between conducive and suppressive soils upon pathogen invasion.

## Materials and Methods

### Soil Sampling

Field soils used in this study were collected from a disease-suppressive field with a consistently low Fusarium wilt disease incidence (15% in 2014) and a nearby disease-conducive field with a high Fusarium wilt disease incidence (70% in 2014) under banana cultivation in Fushan (109.92E and 19.82N), northern Hainan Island, China in 2014. Detailed information on these two fields was provided in our previous study (Shen et al., 2015). Soils were planted with banana in 2015 to confirm the suppressive status in a greenhouse (Supplementary Figure S1). Soil samples were passed through a 2-mm sieve and stored at room temperature. Soils from the suppressive orchard had a pH value of 7.5 and contained 26.93 g/kg of total carbon and 2.16 g/kg of total nitrogen. Soils from the conducive orchard had a pH value of 5.1 and contained 20.03 g/kg of total carbon and 1.58 g/kg of total nitrogen.

### Experiment Description

Two experiments were conducted in this study. The first experiment was carried out in October 2014, and four treatments were established with the inoculation of Foc to a (1) disease-suppressive soil, (2) disease-conducive soil, (3) sterilized disease-suppressive soil, and (4) sterilized disease-conducive soil. Non-sterilized soil samples were preincubated at room temperature in the dark for 2 weeks. Meanwhile, for the sterilized treatments, soils were stored in plastic bags and sterilized by gamma ray (60 KGy) irradiation. Sterility was tested by spreading 5 g of the soil from the bag onto the Luria-Bertani agar media for bacteria and fungi using Rose Bengal agar media by the plate-counting method. No bacterial or fungal colonies were observed on plates after incubation for 3 days. Next, a 200 g portion of sterilized or non-sterilized soil was stored in plastic tissue culture bottles (350 ml), and Foc chlamydospores were added with sterile water and then mixed with soil at a spore density of 2 × 10^5^ g^–1^ dry weight with a final moisture level adjusted to 25% field capacity. The number of spores was estimated by a hemocytometer. Three replicates of each treatment were randomly placed and cultured in a 28°C incubator with constant moisture (25% of field capacity). The plastic tissue culture bottle had a 0.22 μm filter membrane to prevent cross contaminations while allowing gas exchange. Ten grams of soil was sampled from each bottle at 3, 12, 21, and 28 days after inoculation. To investigate the difference in microbiota variation in the two soils in response to pathogen invasion and to investigate the interaction of the native microbes and the pathogen, soil DNA was extracted from the non-sterilized treatments. Specifically, soil genomic DNA from each experimental replicate with 0.25 g soil was extracted using the PowerSoil DNA Isolation Kit (Mobio Laboratories, Carlsbad, CA, United States) according to the manufacturer’s instructions.

To further confirm the result and fate of the introduced strain, we carried out the second experiment in April 2018, a Foc strain containing red fluorescent protein (RFP) and hygromycin B resistance cassettes was added to the same four treatments, except that the sterilized treatment was replaced with soils heat-treated at 115°C for 2 h. Three replicates of each treatment were randomly placed and cultured in a 28°C incubator with constant moisture (25% of field capacity). Ten grams of soil was sampled from each bottle at 3 and 28 days after the addition of the inoculant.

### Pathogen Quantification

For the first experiment, the number of *Fusarium* colony forming units (CFUs) was quantified in each sample by the plate-counting method with modified Komada’s selective medium according to Sun et al. (1978). Briefly, the medium contained 1 g K_2_HPO_4_, 0.5 g MgSO_4_ 7H_2_O, 0.5 g KCl, 0.01 g Fe-Na-EDTA, 20 g D-galactose, 2 g L-Asparagine, and 16 g of agar in 1 L distilled water. In addition, 0.9 g PCNB (pentachloronitrobenzene, 75% WP), 0.5 g Na_2_B_4_O_7_⋅10H_2_O, 0.45 g oxgall, and 0.3 g streptomycin sulfate were added and the pH was adjusted to 3.8 ± 0.2 with 10% phosphoric acid. Another 300 μg ml^–1^ hygromycin B was added to modified Komada’s selective medium to select for the introduced Foc in the second experiment. Plates were cultured at 28°C for 3 days before counting. Quantitative PCR (qPCR) amplification for the determination of the abundances of *F. oxysporum* were performed in non-sterilized soil samples in the first experiment with primers FOF1/FOR1 (Jiménez-Fernández et al., 2010; Lin et al., 2012). A 20-μl total volume PCR was carried out with 10 μl SYBR^®^ *Premix Ex Taq*^TM^ II (Takara Biotechnology Co., Ltd., Dalian, China), 0.8 μl each primer (10 μM), 0.4 μl ROX Reference Dye II (50×), 2 μl template DNA, and 6 μl sterile water. Three replicate reactions were performed on a 7500 Real-Time PCR System (Applied Biosystems, United States) with the following thermal profile: 30 s at 95°C followed by 40 cycles of 5 s at 95°C, and 34 s at 65°C. Standard curves were generated as previously described (Fu et al., 2017). Gene copy numbers were calculated according to standard curve equations.

### Sequencing Library Construction and Sequence Analysis

Soil DNA was subjected to MiSeq sequencing at the Personal Biotechnology Co., Ltd (Shanghai, China). Sequencing libraries were constructed as described previously (Caporaso et al., 2012; Kozich et al., 2013). Briefly, the V4 domain of bacterial *16S rRNA* genes was amplified using primers 520F (5′-AYTGGGYDTAAAGNG-3′) and 802R (5′-TACNVGGGTATCTAATCC-3′) (Claesson et al., 2009). The fungal internal transcribed spacer (ITS) 1 region was amplified using primers ITS1F (5′-CTTGGTCATTTAGAGGAAGTAA-3′) (Gardes and Bruns, 1993) and ITS2R (5′-GCTGCGTTCTTCATCGATGC-3′) (White et al., 1990). Both bacterial and fungal raw reads were removed from the adaptors and primer sequences and then assembled for each sample based on the unique barcode by QIIME V1.9 (Caporaso et al., 2010). Split sequences were merged using FLASH V1.2.7 (Magoč and Salzberg, 2011). Sequences for each sample were processed according to the UPARSE pipeline (Edgar, 2013). Briefly, low-quality sequences were discarded with singletons, removal of errors >0.5 and length <200 (Schöler et al., 2017; Vestergaard et al., 2017). After the removal of chimeric reads (Edgar et al., 2011), OTUs were defined at a 97% similarity identity level. Finally, each representative sequence was classified at 60% confidence by the RDP classifier (version 11.5) (Wang et al., 2007) for bacteria, while fungal sequences were classified using the UNITE database (version 7.2) (Unite Community, 2017) using the naive Bayesian classifier in Mothur (version 1.25.0) (Schloss et al., 2009). Sequences were randomly subsampled to 229,068 reads per sample for bacterial *16S rRNA* gene sequences, while ITS sequences were subsampled to a depth of 62,519 sequences per sample. Phylogenetic trees were constructed using Clearcut in Mothur. Within-sample (alpha) diversity metrics were analyzed by richness (sobs), Pielou’s evenness, and Shannon diversity using Mothur. Weighted UniFrac distance matrixes (Lozupone and Knight, 2005), based on phylogenetics, were generated, and principal coordinate analysis (PCoA) ordinations were performed to explore changes in the composition of the bacterial and fungal communities (beta diversity) between soil samples in Mothur. Multiple regression tree (MRT) analysis (De’Ath, 2002) was calculated using the mvpart (De’Ath, 2007) and MVPARTwrap package (Ouellette and Legendre, 2012) (with default parameters) to relate normalized genus relative abundance data (decostand function) with soil suppressive status and days since inoculation in R software (version 3.3.0) (R Core Team, 2000).

### Relative Abundance Analyses, Network Construction and Analyses

To remove the poorly represented data, both relative abundance analyses and network construction used only the OTUs with average relative abundances >0.01% of the total abundance in at least one time point. Correlation networks of disease-suppressive and disease-conducive soil samples were constructed by OTU abundances across four time points (12 samples for each). For missing data, blanks were replaced with “0.01,” then correlations were calculated using the Sparse Correlations for Compositional data algorithm (SparCC) implemented by python scripts with five iterations. Two-sided pseudo *p*-values were calculated based on bootstrapping of 100 repetitions (Friedman and Alm, 2012). Network was constructed with strong | *r*| > 0.8 and *P* < 0.01 correlations. The potential importance of OTUs (nodes) in the network were usually described with *Zi* (within-module connectivity) and *Pi* (connectivity among modules) (Guimera and Amaral, 2005), which were calculated by rnetcarto packages (Doulcier and Stouffer, 2015) in R. The whole network was divided into four categories (Olesen et al., 2007): (i) peripherals with *Z*i < 2.5 and 0 < *P*i < 0.62; (ii) module hubs with *Z*i ≥ 2.5 and *P*i < 0.62; (iii) connectors with *Z*i < 2.5 and *P*i ≥ 0.62; and (iv) network hubs with *Z*i ≥ 2.5 and *P*i ≥ 0.62. Other topological properties of the empirical networks were computed and visualized by gephi (version 0.9.1) (Bastian et al., 2009) and cytoscape (version 3.4.0) (Shannon et al., 2003).

### Statistical Analysis

Permutational multivariate analysis of variance (PERMANOVA, adonis function, 999 permutations) was used to assess significant differences in soil community composition with permutational analysis of multivariate dispersions (PERMDISP, Beta-disper function, 999 permutations) utilized to confirm equal dispersion between treatments (Anderson, 2001, 2006). Both PERMANOVA and PERMDISP were carried out in the vegan package (Oksanen et al., 2013) based on weighted UniFrac distance matrixes in R. Two-way repeated-measures analysis of variance tests (ANOVA) with Greenhouse-Geisser corrections (when sphericity test was violated) were conducted (non-normal data were square-root or log transformed) in the statistical software package (SPSS 20.0, IBM, New York, NY, United States). Differences in OTU relative abundances between time points were analyzed in R followed by repeated-measures Friedman’s tests with subsequent *post hoc* analyses in the agricolae package (McCrum-Gardner, 2008; De Mendiburu, 2014). All correlations between targets and days since inoculation were calculated using the spearman (within-sample (alpha) diversity with log transformed data) method in R. All trend lines were fitted using geom_smooth with lm function in ggplot2 (Wickham, 2016).

### Accession Numbers

Sequences and sample information were deposited in the NCBI Sequence Read Archive (SRA) database under accession number PRJNA404015.

## Results

### Microbes Harbored in Disease-Suppressive Soil Inhibit Disease-Causing *Fusarium* Growth

**FIGURE 1:**
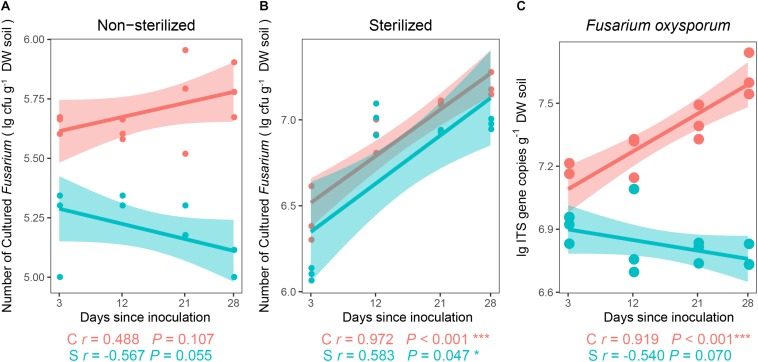
Spearman correlations (*r*) between target and days since inoculation. Number of cultured *Fusarium* in **(A)** non-sterilized treatment, **(B)** sterilized treatment, and **(C)** abundance of *Fusarium oxysporum* in non-sterilized treatments (*n* = 3). C, red color, disease-conducive soil; S, blue color, disease-suppressive soil. The asterisk denotes a statistically significant Spearman correlation (^∗^*P* < 0.05 and ^∗∗∗^*P* < 0.001).

For the first experiment, after the inoculation of Foc into soils for 28 days, colonization abundance of *Fusarium* significantly varied among soils (*P*_Kruskal__–__Wallis test_ = 0.015). In the non-sterilized treatments (Figure 1A), the abundance of cultured *Fusarium* decreased (*r* = −0.567, *P* = 0.055) in the disease-suppressive soil (S) over time but increased non-significantly (*r* = 0.488, *P* = 0.107) with time in the disease-conducive soil (C). In the sterilized treatments (γ-radiation) (Figure 1B), the abundance of the *Fusarium* population increased significantly in both the S (*r* = 0.583, *P* = 0.047) and C (*r* = 0.972, *P* < 0.001) treatments.

The qPCR data showed that the interaction of suppressive status (Soil) and days since inoculation (DAY) (*F* = 8.269, *P* = 0.003; two-way repeated-measures ANOVA) exhibited a significant effect on the abundances of *F*. *oxysporum.* Further analyses showed that there was no observed difference in abundances between the suppressive and conducive soils at day 3 and day 12 (*P*_unpaired_ *_*t*_*_–test_ > 0.05, Figure 1C), although significantly lower abundances were observed in the S compared to C at days 21 and 28 (*P*_unpaired_ *_*t*_*_–test_ < 0.05, Figure 1C).

Although no significant (*P* > 0.05) relationship was observed between the relative abundances of OTUs shown to be classified as Foc (Supplementary Figures S2A,B) and days since inoculation in the two soils (Supplementary Figure S2C), significantly (*P*_Friedman’s test_ < 0.05) lower relative abundances were identified at day 28 compared with day 3 in the disease-suppressive soil.

In the second experiment, the culturable Foc inoculant counts exhibited a variation between the sterilized and non-sterilized suppressive soils after Foc invasion but increased in both the sterilized (heat-treated) and non-sterilized conducive soils (Supplementary Figure S3). The interaction of soil suppressive status, days since inoculation and sterilization status had significant effects on Foc colonization (ANOVA, *P* = 0.01, Supplementary Table S1). Introduced Foc culturable counts were highest in the sterilized suppressive soils and lowest in non-sterilized suppressive soil (*post hoc* pairwise *t*-test, *P*_fdr_ < 0.05).

### Topological Properties of the Networks

**FIGURE 2:**
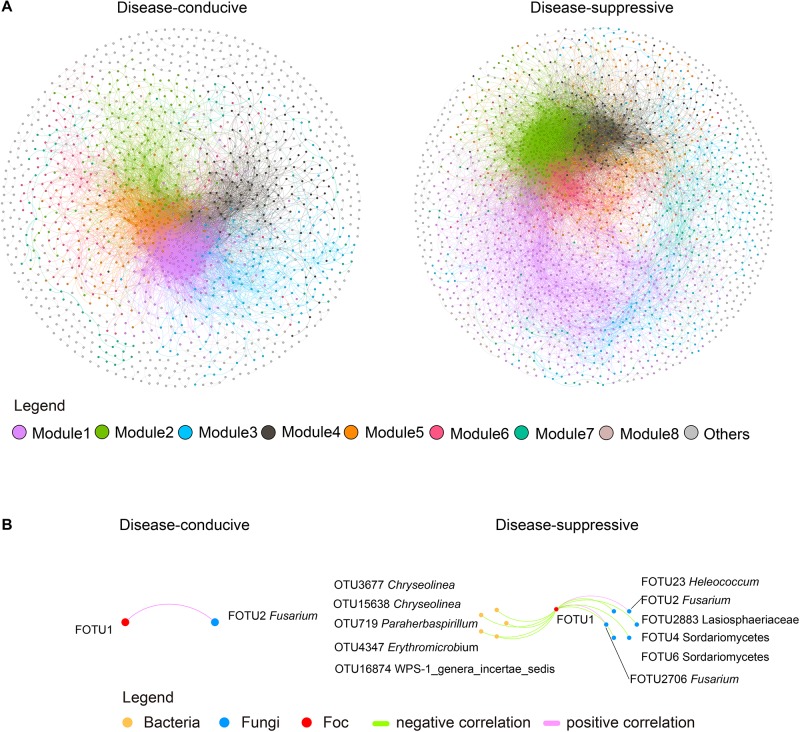
Co-occurrence networks of disease-conducive and -suppressive soils with *F. oxysporum* f. sp. *cubense* (Foc) invasion. **(A)** Network constructed with strong (SparCC’s | *r*| > 0.8) and significant (*P* < 0.01) correlations. Nodes represent different OTUs and are colored by modularity class (fast unfolding) in gephi. Gray color refers to modules with less than 50 nodes. **(B)** OTUs related to Foc OTUs in the co-occurrence network. Each node refers to a unique OTU. Orange, blue, and red represent bacteria, fungi and Foc, respectively. Green and pink edges refer to significant (*P* < 0.01) negative and positive correlations between nodes.

The suppressive soil microbiome formed a larger (with a greater number of nodes) and more complex (with a higher number of linkages) co-occurrence network than the conducive soil (Figure 2A and Supplementary Table S2). The suppressive soil exhibited larger clustering coefficients that indicate closer relationships between nodes and their neighbors, as well as higher modularity, a proposed network division into quasi-functional guilds (Newman, 2003; Supplementary Table S2). Overall, there were 11 unique OTUs in suppressive soil and 1 unique OTUs in conducive soil that were strong (|*r|* > 0.8) and significantly (*P* < 0.01) related to OTUs identified as Foc (Figure 2B and Supplementary Figures S2A,B). In the suppressive network, a larger proportion of negative correlations was observed between Foc and bacterial OTUs (45.45%) vs. fungal OTUs (36.36%).

On the other hand, according to the thresholds of *Zi* (within-module connectivity) and *Pi* (connectivity among modules) (Supplementary Figure S4), one module hub (important to the connection of its own module) in the disease-conducive soil (OTU179, *Gp1*), and nine module hubs in the disease-suppressive soil (OTU3100, *Sterolibacterium*; OTU16004, *Denitratisoma*; OTU1113, *Basilea*; OTU1784, *Sulfuritalea*; OTU16785, *Terrimonas*; OTU11415 *Terrimonas*; OTU1134, *Flavitalea*; OTU10299, *Gp6*; OTU15811, *Flavisolibacter*) were observed. Two connectors (important to network connection) in disease-conducive soil (OTU3146, *Variibacter*; OTU1211, *Acidisoma*) and two connectors in disease-suppressive soil (OTU6129, *Phaselicystis*; OTU4472, *Basilea*) were observed.

### Impact of Pathogen Invasion on Bacterial and Fungal Diversity

**FIGURE 3:**
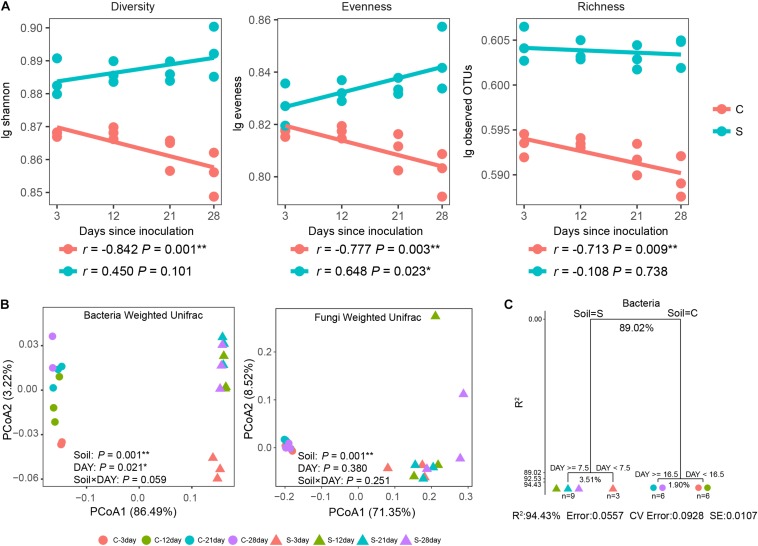
Alpha diversity illustrates different trends between treatments with shifts in community composition since disease-causing *Fusarium* inoculation. **(A)** Spearman correlations (*r*) between bacterial alpha diversity and days since inoculation. Statistical tests are provided in Supplementary Table S2. **(B)** PCoA clusters of the bacterial and fungal community composition based on Weighted UniFrac distance metrics. **(C)** Multiple regression tree analysis of the impacts of soil suppressive status and days since inoculation on bacterial community composition at the genus level. Numbers under the crosses of each split indicate percentages of variance explained. Points represent individual samples, and colors indicate days since inoculation. C, circles, disease-conducive soil; S, triangles, disease-suppressive soil. An asterisk denotes a statistically significant correlation (^∗^*P* < 0.05 and ^∗∗^*P* < 0.01).

Both soil suppression status and days since inoculation exhibited significant (*P* < 0.05) influences on bacterial alpha diversity (Supplementary Table S3). Furthermore, significant (*P* < 0.05) negative correlations were observed between DAY and disease-conducive soil bacterial richness, evenness and diversity together with a significant (*P* < 0.05) positive correlation between DAY and disease-suppressive soil bacterial evenness (Figure 3A). For fungal alpha diversity, evenness and diversity were significantly (*P* < 0.05) impacted by suppressive status (Supplementary Table S3). Meanwhile, no significant relationships between fungal alpha diversities and DAY were observed (Supplementary Figure S5A).

### Shifts in Bacterial and Fungal Communities

A separation between suppressive and conducive bacterial and fungal communities was evidenced by PCoA ordination (Figure 3B), but only the bacterial community composition clearly shifted over time. Both suppression status and days since inoculation resulted in significant (*P*_ADONIS_ < 0.05, *P*_Beta–disper_ > 0.05) differences in soil bacterial community composition. For the fungal community, although samples from the two soils differed from each other in composition (*P*_ADONIS_ = 0.001, *P*_Beta–disper_ < 0.001), no detectable time-related changes in the soil fungal community were identified (*P*_ADONIS_ > 0.05, *P*_Beta–disper_ > 0.05). The influence of the large Foc population was further investigated by recalculating the fungal community composition, since they accounted for more than 30% of the relative abundance in the conducive soil (Supplementary Figure S2C). These modified weighted UniFrac distance matrixes, recalculated after the removal of Foc sequences and their related OTUs, yielded similar results, indicating that the large Foc proportion did not significantly (*P* < 0.05) influence the results (Supplementary Figure S5C).

Relationships between community composition at genus level and both soil suppression status and days since inoculation were evaluated by MRT analysis. For bacteria, a best-prediction tree with four terminal nodes was observed (Figure 3C) that explained 94.43% of the original variance. For fungi, the best prediction tree was split into two groups by soil with 58.3% of the original variance explained (Supplementary Figure S5B). These results suggest that the soil bacterial but not the fungal community changed over time. Although suppressive status explained the majority (*R*^2^ = 89.02%) of the complexity in the data (Figure 3C), the passage of time after inoculation accounted for more variation in the suppressive soil subgroups (*R*^2^ = 3.51%) than in the conducive soil subgroups (*R*^2^ = 1.90%). Terminal nodes indicated that the suppressive soil bacterial genera initiated an earlier response (Figure 3C).

**FIGURE 4:**
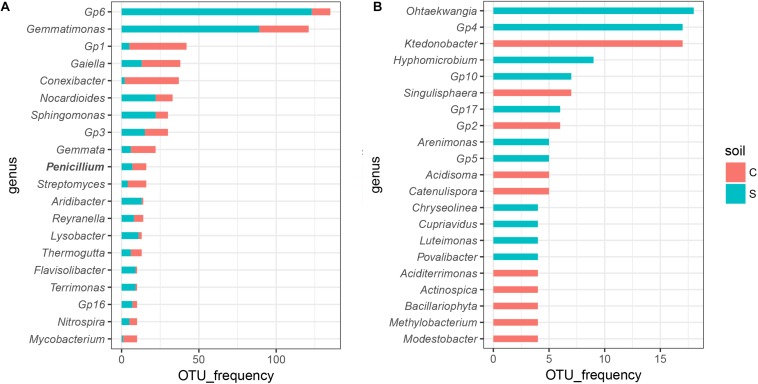
Sensitive OTUs that responded to invading *Fusarium* varied by soil suppression status. Significantly altered (*P*_Friedman’s test_ < 0.05) OTUs in at least one time point in the disease-suppressive and disease-conducive soils classified to the genus level. Bar plots show the top 20 **(A)** shared genera and those that were **(B)** unique to one soil.

We further analyzed which group drove the compositional shifts upon invasion. In general, the microbial response was highly impacted by soil suppression status after Foc invasion (Supplementary Figure S6). Among the calculated OTUs, in the conducive soil, 51.67% of bacterial OTUs and 21.25% of fungal OTUs were significantly altered (*P* < 0.05) at least one time point after invasion, while for the suppressive soil, 54.27% of bacterial OTUs and 25.04% of fungal OTUs were changed (Supplementary Table S4). Only 119 bacterial (6.33% for conducive soil and 5.18% for suppressive soil) and 14 fungal (2.50% for conducive soil and 2.34% for suppressive soil) OTUs were shared between the two soils, occupying a small proportion in the total calculated OTUs. Those OTUs that significantly (*P* < 0.05) changed in relative abundance were then classified into the genus level, and the disease-suppressive soil harbored more OTUs in *Gp6*, *Gemmatimonas*, *Nocardioides*, *Sphingomonas*, *Aridibacter*, *Reyranella*, *Lysobacter*, *Flavisolibacter*, and *Terrimonas* than disease-conducive soil (Figure 4A). *Othaekwangia*, *Gp4*, *Hyphomicrobium*, *Gp10*, *Gp17*, *Arenimonas*, *Gp5*, *Chryseolinea*, *Cupriavidus*, *Luteimonas*, and *Povalibacter* were the unique genera that significantly (*P* < 0.05) changed in disease-suppressive soil (Figure 4B).

**FIGURE 5:**
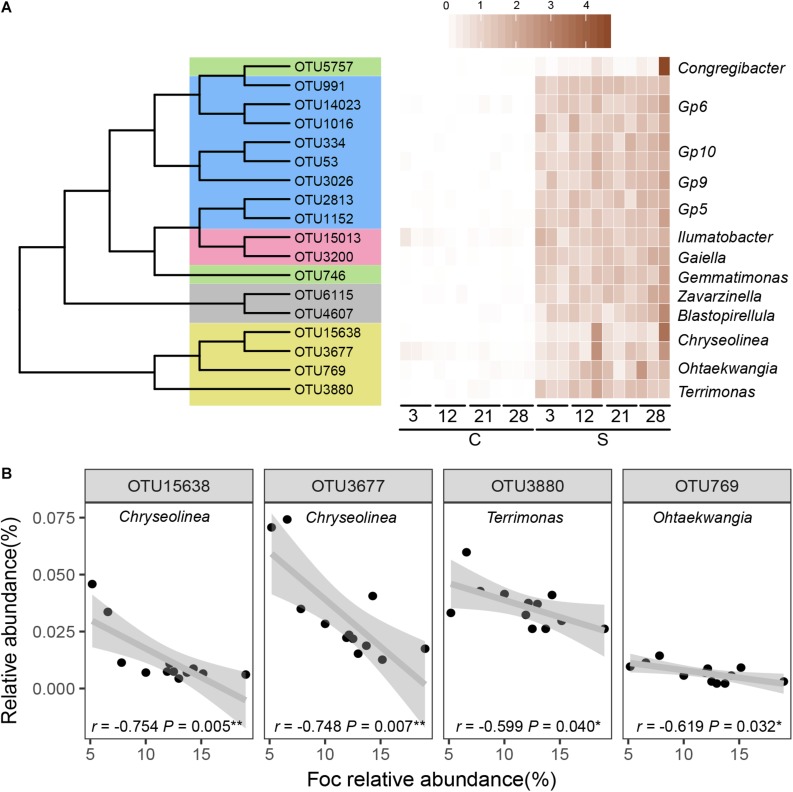
Key OTUs contributed most to the suppressive microbiome. **(A)** Heatmap displaying the OTUs (with RA > 0.01% at least one time point) significantly negatively (*P*_spearman_ < 0.05) correlated with Foc in the suppressive soil based on relative abundance together with matches with an RDP confidence estimate above 0.6 at the genus level. **(B)** Spearman correlations (*r*) between relative abundances (RA) of taxa with biocontrol capacity and *Fusarium*.

The relative abundances of OTUs that were significantly (*P* < 0.05) negatively correlated with Foc in the suppressive soil over the course of the experiment were assumed to play important roles in resistance to Foc invasion. A total of eighteen OTUs (Figure 5A) were identified as such to thirteen bacterial genera (*Blastopirellula*, *Chryseolinea*, *Congregibacter*, *Gaiella*, *Gemmatimonas*, *Gp5*, *Gp6*, *Gp9*, *Gp10, Ilumatobacter, Ohtaekwangia, Terrimonas*, and *Zavarzinella*).

## Discussion

In this study, we showed a differential temporal shift in the soil microbiome in the presence of *F. oxysporum* f. sp. *cubense* (Foc) invasion between suppressive and conducive soils. The soil microbiome is known to play an important role in modulating plant diseases (Mendes et al., 2011; Pieterse et al., 2014; Lebeis et al., 2015). The current study sheds light on potentially underlying agents in disease suppressive soils and provides a good model to understand the microbial consortia and mechanisms involved in disease suppression.

After soil sterilization (γ-radiation and heat-treated), different abiotic factors in the two soils did not inhibit the *Fusarium* increase (Figure 1 and Supplementary Figure S3). Opposite trends in the number of disease-causing *Fusarium* detected by both cultivable and qPCR methods were only observed in sterilized (γ-radiation and heat-treated) and non-sterilized suppressive soils after pathogen invasion (Figure 1 and Supplementary Figure S3). These results reveal the potential of suppressive soils in inhibiting *F. oxysporum* by the soil microbiome as described by Weller et al. (2002), and the suppressive soil microbiome is directly antagonizing the invading pathogen, resulting in lower abundances over time, which is in contrast to the conducive soil. Moreover, abiotic factors have been reported to inhibit fungistasis through the microbiome, and previous research observed that acidification increased Actinobacterial abundances in soil, which subsequently suppressed the pathogen (Trivedi et al., 2017; Dai et al., 2018). However, in this study, regardless of sterilization, the conducive soil with pH < 7 did not reveal pathogen suppression ability.

Microbial-mediated disease suppression is thought to occur through two principal mechanisms: (a) the activities of a metabolically active microbial community and (b) the proliferation of specific microbial agents with anti-pathogen properties (Hadar and Papadopoulou, 2012). The second principal mechanism can occur through four mechanisms: (1) the production of antibiotics or other compounds that are toxic to pathogens (Mendes et al., 2011), (2) competition for nutrients (Elad and Baker, 1985), (3) competition for space on plant tissue for invasion (Pantelides et al., 2009), and (4) predation/parasitism of pathogens by lytic bacteria and fungi (Hoitink and Boehm, 1999; Öztopuz et al., 2018). To putatively identify underlying agents of soil suppressiveness, network co-occurrence analysis was used to reveal complex interactions within microbial communities through competition or cooperation for nutrients, carbon substrates, and space (Deng et al., 2012). In this study, as the ITS region could not distinguish fungi effectively at the species level (Nilsson et al., 2019), two fungal OTUs were identified, similar to Foc pathogens. However, with strong (| *r*| > 0.8) and significant (*P* < 0.01) correlations, only one of them existed in the network. Topological properties describing these complex relationships suggest that the disease-suppressive soil network exhibits more complex interactions between microorganisms. In addition, co-occurrence relationships between microbial OTUs and Foc OTUs varied with suppression status. Specifically, the disease-suppressive soil network exhibited a higher number of co-occurrence links with Foc than the disease-conducive soil network (Figure 2B). The higher number of both positive and negative interactions suggests a greater degree of microbial cooperation and competition, respectively (Zhang et al., 2014; Xiong et al., 2017). Bacterial OTUs exhibited negative linkages with Foc in the disease-suppressive soil network. This effect may be caused by direct antibiosis between bacterial OTUs and Foc (Mendes et al., 2011; Cha et al., 2016), leading to limitations on Foc growth and indicating that the bacterial community may be more involved in the development and maintenance of a disease suppressive status in these soils. Bacteria had a redundant effect on fungi, and antibiotic-resistance genes from a global study revealed an antagonistic relationship between fungi and bacteria (Bahram et al., 2018; Duran et al., 2018). Meanwhile, antagonism bacteria added to soil can reduce Fusarium wilt disease (Wang et al., 2013). Although non-pathogenic *F. oxysporum* could suppress Fusarium wilt of banana (Nel et al., 2006), in this study, non-pathogenic *F. oxysporum* showed a positive correlation with Foc (Figure 2B) in the network, suggesting that non-pathogenic *F. oxysporum* did not show suppression ability in this study.

Microbial diversity has been thought to be linked with soil suppression (Garbeva et al., 2004), as higher microbial diversity might also increase functional diversity (Van Der Heijden et al., 2008; Delgado-Baquerizo et al., 2016). Our results show that although the same amount of Foc spores was added to the conducive and suppressive soils, higher bacterial evenness, richness, diversity, and fungal evenness and diversity were identified in the suppressive soil (Figure 3A and Supplementary Figure S5A). Previous studies revealed that a higher diversity of banana varieties, clay content, pH and lower soil cover by graminoids may contribute to soil disease suppressiveness to Foc race 1 (Deltour et al., 2017), and disease severity caused by inoculation of Foc race 1 was negatively correlated with soil clay content and β-glucosidase activity (Bowen et al., 2019). In this study, we observed microbial diversity linked to pathogen suppression. A similar result of higher bacterial diversity was found in potato scab suppressive soil (Rosenzweig et al., 2012) and higher fungal richness in vanilla Fusarium wilt suppressive soils than in conducive soils (Xiong et al., 2017). High microbial richness is thought to be a key element for the suppression of soil-borne disease because resources should be utilized more efficiently (Loreau and Hector, 2001), thereby preventing the establishment of a pathogen within an empty niche. Previous studies also found that antagonistic interactions increased with species richness (Becker et al., 2012), and higher bacterial diversity hindered pathogen establishment in soil (Dey et al., 2012; van Elsas et al., 2012).

Although fungi may have played a role in preventing *F. oxysporum* infection of vanilla in suppressive soils (Xiong et al., 2017), in this study, only the composition of the bacterial community was significantly altered over time after Foc inoculation (Figure 3B). The Foc inoculum accounted for more than 30% of the total fungi relative abundance (Supplementary Figure S2C) in the conducive soil and remained quasi-stable over time. It was thus suspected that the contribution from the Foc OTUs may influence the ordination of the overall community and comparisons between C and S treatments. As such, we removed those OTUs from the analysis and found that comparisons of the fungal community composition did not change, with significant differences remaining between the treatments (Supplementary Figure S5C). Previous studies have also shown that suppressive and non-suppressive fungal communities exhibited significant overall differences in composition (Penton et al., 2014; Xiong et al., 2017).

For the bacterial community, significant differences in community composition between conducive and suppressive soils were also previously identified (Kyselková et al., 2009; Mendes et al., 2011). Although time influenced bacterial community composition in both soils (Figure 3A), MRT analysis revealed that changes in bacterial communities appeared earlier in suppressive than in conducive soils (Figure 3C). The results could be explained by the reduced performance of the pathogen in species-rich vs. species-poor bacterial communities partly due to competition for space (Bolwerk et al., 2003) and resources (Wei et al., 2015). Our results specifically indicate that the rapid change in bacterial composition may be the primary mechanism of suppression in these soils and with this specific pathogen.

A larger number of OTUs responded significantly to invading *Fusarium* in the suppressive soil, while in both soils, more bacterial OTUs responded to invading *Fusarium* than fungal OTUs. OTUs that negatively (*P* < 0.05) correlated with Foc also differed between suppressive and conducive soils (Figure 5A and Supplementary Figure S7). Although a specific suppressive function cannot be directly attributed to these “sensitive” OTUs in disease-suppressive soil, several taxa are reported to have documented biocontrol capacities. In our results, we found that OTUs belonging to the *Chryseolinea*, *Ohtaekwangia*, and *Terrimonas* were negatively correlated with Foc relative abundance (Figure 5B) in disease-suppressive soil. *Terrimonas*, *Ohtaekwangia*, and *Chryseolinea* were also the dominant genera among the significantly changed OTUs in disease suppressive soil compared to disease-conducive soil (Figure 4). Meanwhile, OTUs assigned to *Terrimonas* were two of the module hubs, and OTUs assigned to *Chryseolinea* were significantly negative with Foc in the disease-suppressive network (Figure 2B and Supplementary Figure S4). This finding indicates that these bacteria may play an important role in the prevention of Foc establishment in disease-suppressive soil. Current knowledge concerning *Terrimonas*, *Ohtaekwangia* and *Chryseolinea* is limited, although species within the Chitinophagaceae family and Cytophagales order are known to produce chitinolytic enzymes (Reichenbach, 2006; Glavina et al., 2010). These enzymes enable lysis of fungal cell walls (Singh et al., 1999). There were *Blastopirellula*, *Congregibacter*, *Gaiella*, *Gemmatimonas*, *Gp5*, *Gp6*, *Gp9*, *Gp10*, *Ilumatobacter*, and *Zavarzinella* that were linked with Foc, but we cannot explain this link ecologically to date. Overall, our results suggest that Foc invasion into a suppressive soil changes the relative abundances of aforementioned “sensitive” OTUs (significantly negatively correlated with Foc). These taxa likely reduce pathogen abundances, which affects the overall microbial community composition and function.

## Conclusion

The colonization of disease-causing *Fusarium* varied by soil suppression status, with naturally disease suppressive soils to Fusarium wilt disease of banana being confirmed to suppress the survival of the disease-causing *Fusarium* over time. Our study revealed that microbiome connectedness was higher in suppressive than conducive soils to Fusarium wilt disease. Furthermore, we determined the potential drivers of suppressiveness that we believe are found among bacteria. Of the key taxa identified in this study, *Chryseolinea*, *Terrimonas*, and *Ohtaekwangia* were the most conspicuous bacteria that appear to contribute to suppression. Therefore, the results of this study may guide future efforts in targeted cultivations and experimentation to evaluate the potential biocontrol potential of these taxa, which could lead to an effective biocontrol strategy that can be widely applicable to fight against the highly prominent and widespread *Fusarium* pathogen.

## Data Availability Statement

The datasets generated for this study can be found in the NCBI Sequence Read Archive (SRA) database under accession number PRJNA404015.

## Author Contributions

RL, ZS, QS, and YO conceived and designed the experiments. YS and NL performed the experiments. YO analyzed the data. YO, RL, ZS, BW, YR, and QS contributed reagents, materials, and analysis tools. YO, RL, CP, SG, and WX wrote the manuscript.

## Conflict of Interest

The authors declare that the research was conducted in the absence of any commercial or financial relationships that could be construed as a potential conflict of interest.
