# Supplementary material for: Deciphering Underlying Drivers of Disease Suppressiveness Against Pathogenic Fusarium oxysporum
Source: Front Microbiol. 2019 Nov 12;10:2535. doi: 10.3389/fmicb.2019.02535 (PMC6861331; doi:10.3389/fmicb.2019.02535)
Supplement: Supplementary file 1 [file Data_Sheet_1.pdf]

Supplemental material

Fig. S1 Fusarium wilt disease incidence after two seasons. Banana plants were both planted with new banana seedlings of two seasons in greenhouse (from February 2015 to November 2015). S and C stand for disease-suppressive and -conducive soils, respectively. Error bars represent s.e. (n = 4). Differences were detected by unpaired Wilcox test (*$P < 0.05$).

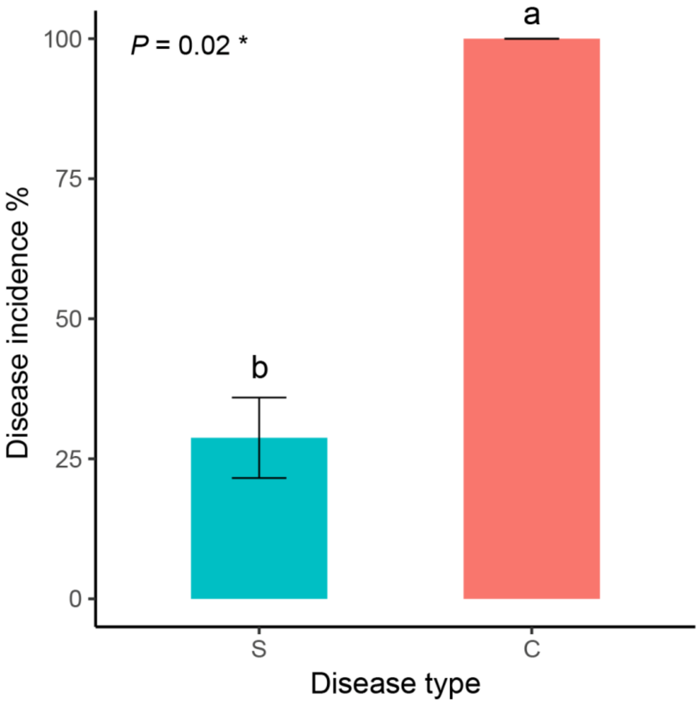

Fig. S2 *Fusarium oxysporum* f. sp. *cubense* identification and the distribution of its relative abundances. (a) OTUs classified as *Fusarium* genus were aligned together with *Fusarium oxysporum* f. sp. *cubense* (Foc) isolate EPPI01 (EU022522.1) and *Fusarium oxysporum* f. sp. *cubense* race 4 (LT571434.1) by muscle, sequences highlighted differences from the Foc4 sequence. (b) Further Maximum-likelihood tree with 1000 bootstrap (MEGA) showed that FOTU1 and FOTU581 had the closest phylogenetic relationship with Foc. (c) Spearman correlations between days since inoculation and relative abundance of Foc. C: red color, disease-conducive soil; S: blue color, disease-suppressive soil.

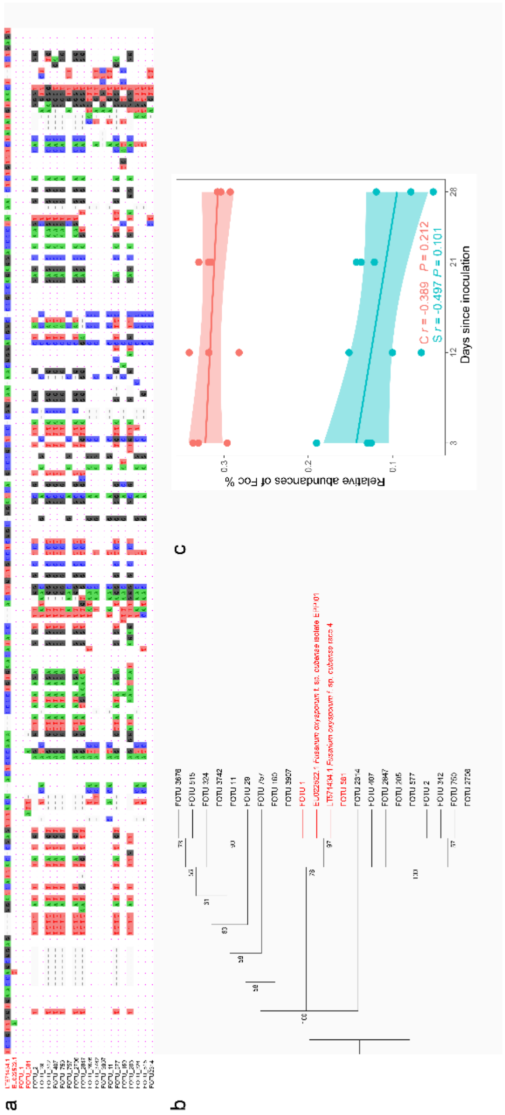

Fig. S3 Microbes harbored in disease-suppressive soil inhibit invading *Fusarium* growth. (a) Number of cultured invading *Fusarium* in nonsterilized treatment and sterilized treatment (n=3); C: red color, disease-conducive soil; S: blue color, disease-suppressive soil. Differences were detected upon the soil suppressive status, days since inoculation, sterilization and their interaction by three-way repeated measures (*$P < 0.05$; **$P < 0.01$; ***$P < 0.001$) (Table S1).

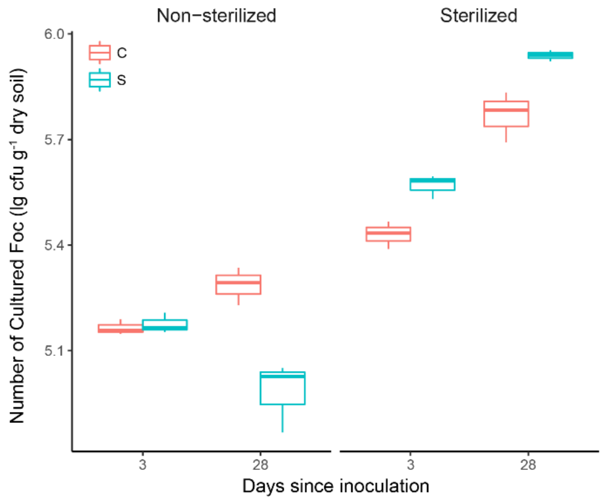

Fig. S4 *Zi-Pi* plot showing the distribution of OTUs based on their topological roles in two soils. Each symbol represents an OTU. OTUs pointed out belonging to four categories.

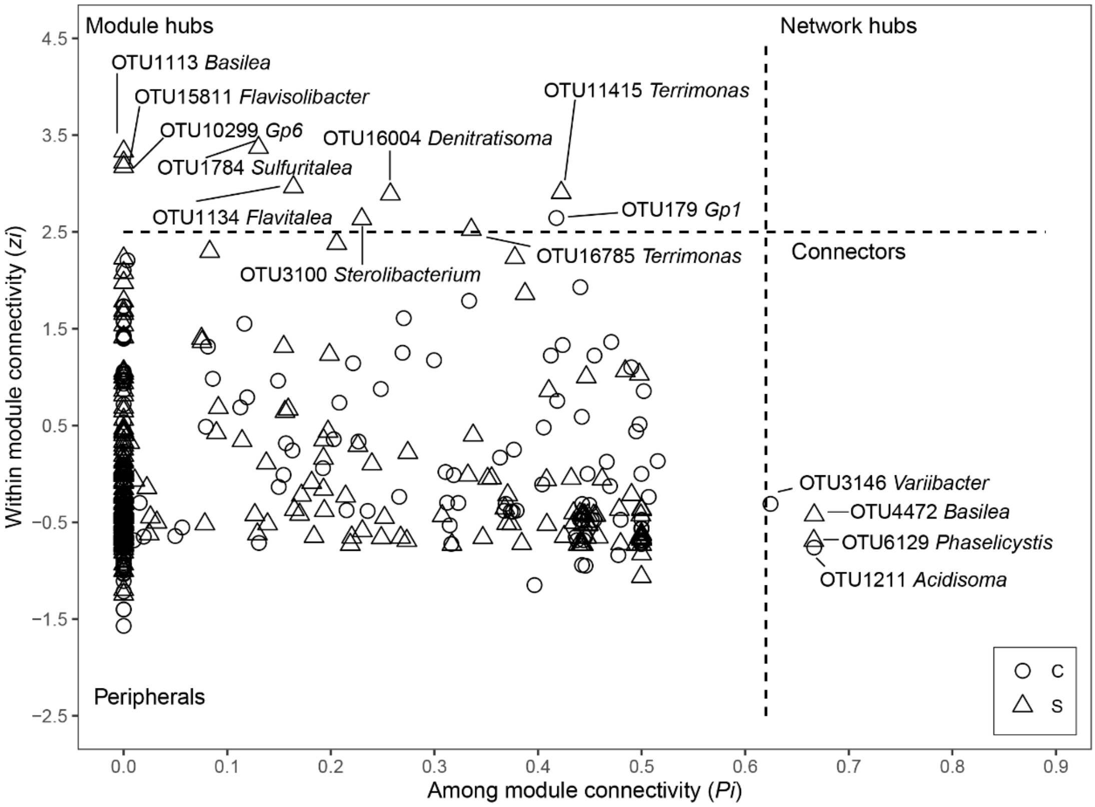

Fig. S5 Fungal alpha and beta diversity. (a) Spearman correlations (*r*) between fungal alpha diversity and days since inoculation. (b) Multiple regression tree analysis of factors impact fungal community composition (genus level). Factors indicate soil suppression status and days since inoculation. Numbers under the crosses of each split indicate percentages of variance explained by the split. (c) PCoA clusters of the fungal without *Fusarium oxysporum* f. sp. *cubense* community composition based on Weighted UniFrac distance metrics; Points represent individual samples and colors indicate days since inoculation; C, disease-conducive soil; S, disease-suppressive soil. Asterisk means a statistically significant (*$P < 0.05$, ** $P < 0.01$)

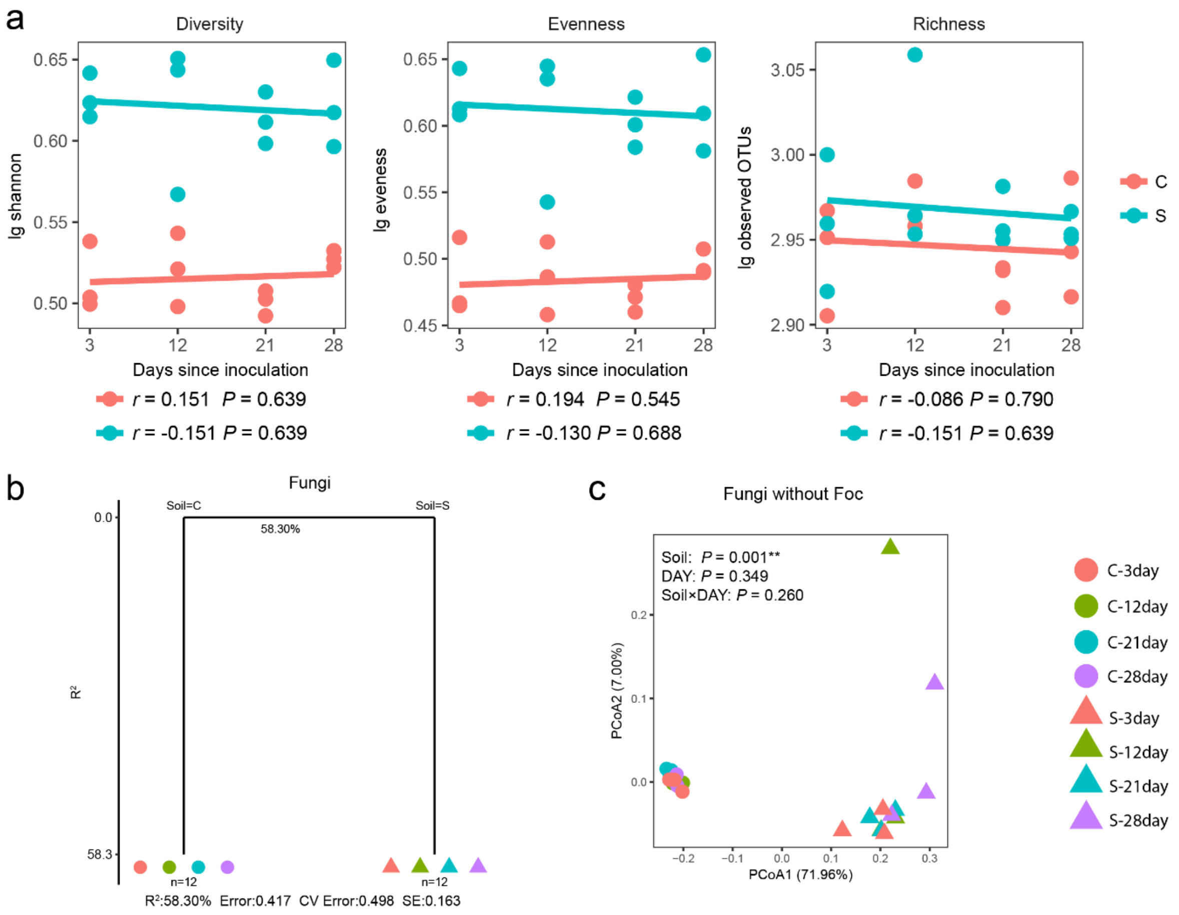

Fig. S6 Sensitive OTUs responded to invading *Fusarium* varied by soil suppression status. Significantly changed ($P_{\text{Friedman's test}} < 0.05$) OTUs in at least one time point in the disease-suppressive and -conducive soil were classified to the phylum level.

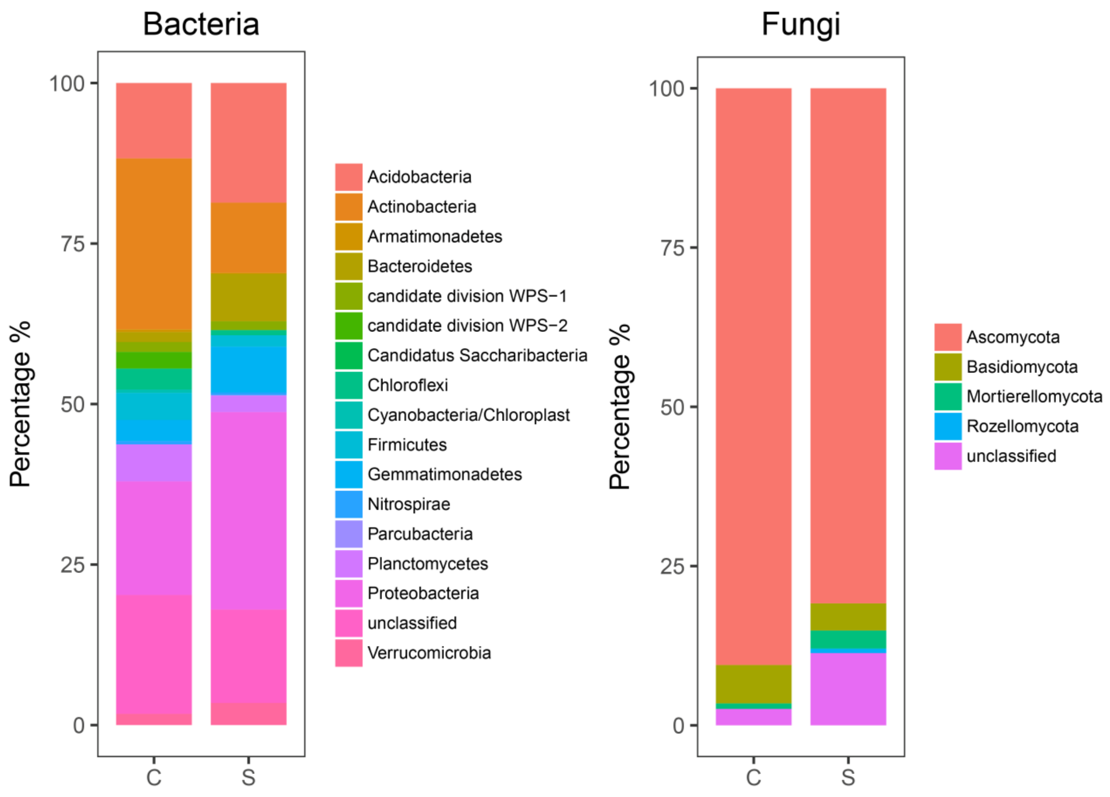

Fig. S7 Heatmap displaying the OTUs (with RA > 0.01% at least one time point) significantly negatively ($P_{spearman}$ < 0.05) correlated with *Fusarium oxysporum* f. sp. *cubense* in the disease-conducive soil based on relative abundance, together with matches with an RDP confidence estimate above 0.6 at the genus level.

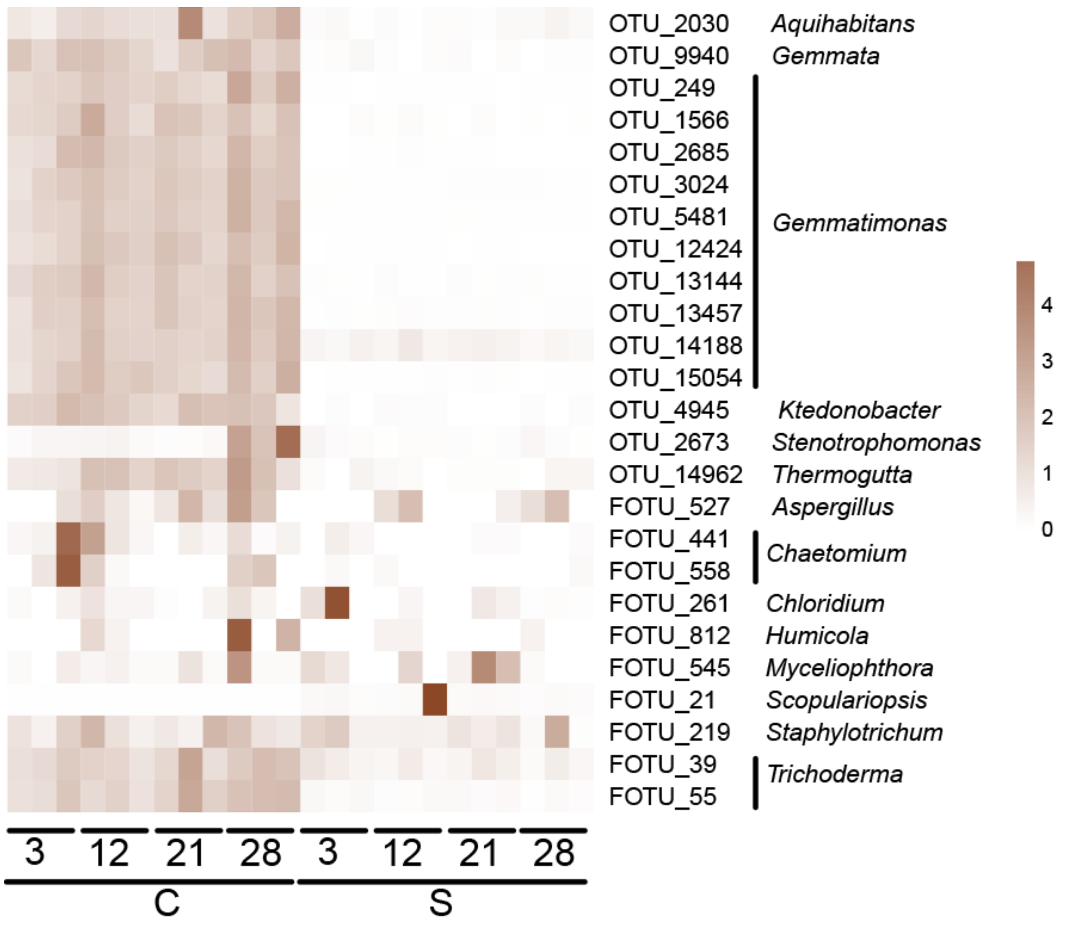

Table S1. Three-way repeated-measures ANOVA for cultured *Fusarium oxysporum* f. sp. *cubense*.

Source of variation	*d.f.*	*F* model	*P*
Soil	1	0.033	0.860
DAY	1	11.330	0.005**
Tre	1	62.395	<0.001***
Soil×DAY	1	11.434	0.005**
Soil ×Tre	1	50.851	<0.001***
DAY×Tre	1	85.751	<0.001***
Soil ×DAY×Tre	1	16.654	0.001**
Residuals	13		

Soil means soil suppressive status, DAY means days since inoculation, Tre means sterilization status. Asterisk indicates a significant difference (*$P < 0.05$, ** $P < 0.01$, *** $P < 0.001$).

Table S2. Topological properties of the empirical networks by gephi (P < 0.01).

Correlation	Suppression status	Total nodes	Total links	Density	Clustering coefficient	Modularity
						Fast Unfolding
$\lvert r \rvert > 0.8$	Conducive	1372	10514	0.011	0.275	0.401
	Suppressive	2013	16683	0.008	0.279	0.492

Table S3. Two-way repeated-measures ANOVA with Greenhous-Geisser correction (when sphericity test was violated) for within-sample (alpha) diversity.

Target	Source of variation	Bacteria			Fungi		
		d.f.	*F* model	*P*	*d.f.*	*F* model	*P*
Richness	Soil	1	49.168	<0.001***	1	0.159	0.697
	DAY	3	3.513	0.049*	3	0.342	0.796
	Soil×DAY	3	0.206	0.890	3	0.058	0.981
	Residuals	12			12		
Eveness	Soil	1	4.900	0.047*	1	57.407	<0.001***
	DAY	3	11.344	0.001**	3	1.931	0.179
	Soil×DAY	3	0.564	0.649	3	2.893	0.079
	Residuals	12			12		
Diversity	Soil	1	20.273	<0.001***	1	59.385	<0.001***
	DAY	3	9.713	0.002**	3	2.867	0.081
	Soil:×DAY	3	0.375	0.773	3	3.028	0.071
	Residuals	12			12		

Soil means soil suppressive status, DAY means days since inoculation. Asterisk indicates a significant difference (*$P < 0.05$, ** $P < 0.01$, *** $P < 0.001$).

Table S4. Number of (A) calculated OTUs (average relative abundance > 0.01% at

least one time point); (B) significantly changed OTUs at least one time point ($p < 0.05$)

	A		B	
	conducive	suppressive	conducive	suppressive
Total	2441	2895	1091	1396
Bacterial	1881	2296	972	1246
Fungi	560	599	119	150
